# Adsorption of Vitamin B12 on Sugarcane-Derived Activated Carbon: Fractal Isotherm and Kinetics Modelling, Electrochemistry and Molecular Modelling Studies

**DOI:** 10.3390/molecules30102096

**Published:** 2025-05-08

**Authors:** Ronald Ranguin, Mohamed Chaker Ncibi, Corine Jean-Marius, François Brouers, Gerardo Cebrián-Torrejón, Antonio Doménech-Carbó, Steffen Souila, José-Emilio Sánchez-Aparicio, Daniel Dorce, Iker Zapirain-Gysling, Jean-Didier Maréchal, Ulises Jauregui-Haza, Sarra Gaspard

**Affiliations:** 1 Laboratory “Connaissance et Valorisation: Chimie des Matériaux, Environnement, Énergie” (COVACHIM–M2E–EA 3592)—Faculté des Sciences Exactes et Naturelles, Université des Antilles, B.P. 250, CEDEX, 97157 Pointe-à-Pitre, France; rranguin@skillcell-alcen.com (R.R.); corine.jean-marius@univ-antilles.fr (C.J.-M.); gerardo.cebrian-torrejon@univ-antilles.fr (G.C.-T.); dorcedaniel1991@yahoo.fr (D.D.); 2 CNRS/SKILLCELL FRE 3690, Sys2Diag, Cap Delta, 1682 rue de la Valsière, 34184 Montpellier, France; 3 International Water Research Institute, Mohammed VI Polytechnic University, Green City Ben Guerir 43150, Morocco; chaker.necibi@um6p.ma; 4 Emeritus Professor of Faculty of Applied Physics, Liege University, 4000 Liège, Belgium; fbrouers@yahoo.fr; 5 Departament de Química Analítica, Universitat de València, Dr. Moliner, 50, 46100 Burjassot, València, Spain; steffen.souila@outlook.fr; 6 InSiliChem, Departament de Química, Universitat Autònoma de Barcelona, Campus Bellaterra, 08193 Cerdanyola-del-Vallés, Barcelona, Spain; joseemilio.sanchez@uab.cat (J.-E.S.-A.); i.z.gysling@gmail.com (I.Z.-G.); jeandidier.marechal@uab.cat (J.-D.M.); 7 Área de Ciencias Básicas y Ambientales, Instituto Tecnolόgico de Santo Domingo (INTEC), Santo Domingo 10602, Dominican Republic; ulises.jauregui@intec.edu.do

**Keywords:** nanomaterials, activated Carbon, vitamin B12, electrochemical, molecular modelling, molecular docking

## Abstract

In the present work, the adsorption of vitamin B12 (VB12) on sugarcane-derived activated carbon (AC) was investigated with the purpose of developing a hybrid material able to degrade highly toxic and recalcitrant chlordecone (CLD) for remediating the severe environmental issue of pesticide pollution of water and soil. The AC used is made from locally accessible sugarcane bagasse. The experimental kinetic and isothermic data of VB12 adsorption on AC were modeled using multiple models, including Pseudo-Order 1 (Lagergren), Pseudo-Order 2, Elovich, and Brouers–Sotolongo models for the kinetics. The isotherms models used were Langmuir, Freundlich, Hill–Sips, Brouers–Sotolongo (BS), Brouers-Gaspard (BG), General Brouers–Sotolongo (GBS), and Redlich–Peterson (RP) models. The results showed that the BG model is the most suitable to satisfactorily describe the adsorption of VB12 on the studied AC, involving a heterogeneous adsorption mechanism onto a heterogeneous surface and providing the maximum adsorption capacity, a convenient tool to estimate the saturation level of adsorbate (i.e., chlordecone (CLD)) onto the adsorbent (AC). Voltammetric studies confirm the interaction between VB12 and the AC. Finally, molecular modeling is used to provide atomic insights showing the entrapment of VB12 inside the porous system to form a new hybrid material. The calculations provide the conformations with the best binding energy in the GaudiMM environment.

## 1. Introduction

Porphyrin non-covalent interactions with carbon materials such as carbon nanotubes, single-walled nanotubes, and multi-walled carbon nanotubes and activated carbon (AC) are of particular interest for the design of new nanomaterials with interesting electronic and optical properties, as well as valuable environmental applications. Non-covalent binding has an advantage as a functionalization process over the covalent techniques of producing stable materials that do not disrupt the electronic, optical, or catalytic properties of both components of the nanohybrid material [1,2]. In the case of environmental applications, the use of AC-supported porphyrin catalysts allows faster degradation of the pollutant but, as well as catalyst fixation to the material surface, allows expensive catalyst saving [3].

The study of adsorption kinetics and equilibrium is critical in providing guidance for the design and procedure of adsorption processes on material surfaces, such as AC, to achieve non-covalent functionalization. Many models have been used to describe the transport of solutes inside the AC pores [4,5,6]. The overall rate of adsorption of organic compounds on AC is the most often interpreted via first [7,8] or second-order kinetics [9]. The rate of adsorption on an active site is thought to be the controlling step, and it can be assumed to follow a first or second-order reaction in these adsorption reaction models. However, it is very well documented in the literature that the overall rate of adsorption in a porous adsorbent must not only take into account the adsorption rate on an active site but also the external mass transfer and intraparticle diffusion [10]. Indeed, solute diffusion in liquid–porous solid systems may be governed by pore volume diffusion, surface diffusion, or a combination of both mechanisms [11]. Adsorption is a complex phenomenon that may also involve chemical interactions between the solute and the chemical groups on the AC surface, involving different types of interactions such as electrostatic, Van der Waals, hydrogen bonding, or hydrophobic interactions. The Elovich model [12] has been applied satisfactorily to describe the chemisorption of adsorbates on sorbents with heterogeneous surfaces. However, it has been shown that the adsorption process of a solute on an AC, which takes place at the liquid–solid boundary, is clearly a “heterogeneous” reaction, and the interface of the two phases represents a special environment under dimensional or topological constraints [13,14]. Therefore, these systems can be associated with the class of complex systems, and adsorption kinetics can be described by a fractal kinetic equation [13,14,15]. Some of our previous works clearly showed that fractal kinetic equations, which have been extensively applied to biophysical phenomena, can be a useful theoretical tool for studying the adsorption processes of single compounds, wastewaters, or drugs on AC [16,17,18,19].

Adsorption isotherm data are classically described using Langmuir or Freundlich equations. These models are empirical and bring little information on the physicochemical processes responsible for a particular shape of the isotherm curve. Brouers has shown that most of these empirical models derive from the Burr–Maddala distribution (BurrXII) [20].

Based on the Weron stochastic theory of relaxation in complex systems, which is related to birth and death models [21]. The Brouers–Sotolongo (BS) equation is a generalization of the first-order kinetic equation, which gives an exponential decay. It includes a fractal time and a non-integer order n [22]. The solution is mathematically a deformed exponential: an extended BurrXII function [23] equivalent to the Singh–Maddala function [24] used in econometrics, sorption theories, and fractal kinetics [18,25].

The Langmuir, Hill–Sips, and Brouers–Sotolongo isotherms were proven to be reliable statistical functions having correct asymptotic limits for fitting adsorption isotherm data at low and high concentrations [26].

Modeling of experimental data from adsorption processes is a very important means for the prediction of the mechanisms of the adsorption.

Classical adsorption kinetic and isotherm models derived from the generalized equation were evaluated and compared for their ability to describe kinetic and isotherm data on ACs accurately. It is proposed to demonstrate the feasibility of using a new methodology for kinetic and isotherm data modeling by comparing the precision of the generalized and derived equations based on their non-linear fitting. Previously published works showed that vitamin B12 (VB12) is able to achieve the degradation of chlordecone (CLD), a persistent pollutant, leading to the opening of the CLD cage structure to produce pentachloroindene [27,28]. This result was confirmed in a study showing the possibility of degrading and adsorbing the persistent organic pesticide, CLD, in a single step using a hybrid material obtained by VB12 adsorption on AC [28]. VB12 is an organocobalt porphyrin synthesized by certain bacteria concentrated in the food chain, which provides the major dietary sources of VB12 in meat and dairy products [29]. This cobalt-containing porphyrin, VB12, is known for its ability to reduce chlorinated pollutants [30,31,32,33].

In this study, the adsorption kinetics of VB12 on AC are studied as a function of different parameters such as AC mass, VB12 concentration, and temperature in order to be able to find the best condition in the studied interval to determine the amount of VB12 that can be adsorbed on the AC [34]. Adsorption isotherm data are also modeled to obtain a better insight into the adsorption mechanism of VB12 on AC [35]. The interactions between AC material and VB12 are also monitored electrochemically, and a molecular modeling study of the formation of this hybrid material is carried out.

## 2. Results

### 2.1. Specific Surface Area and Pore Size

**Figure 1:**
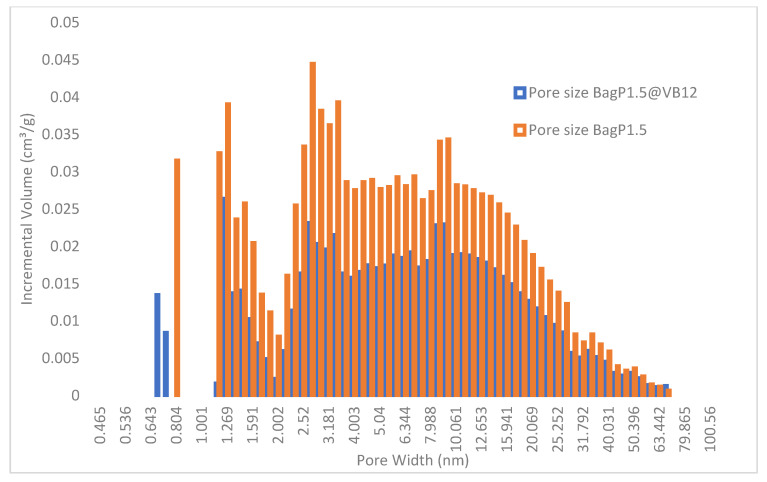
Histograms and pore diameter size distributions for analyzed samples BagP1.5 and BagP1.5@VB12.

The histogram pore size distributions of BagP1.5 and BagP1.5@VB12 samples are displayed in Figure 1. The main features are the reduction in porous volume in micropores and mesopores range. In the micropores range, formation of smaller micropores in the BagP1.5@VB12 sample suggests that VB12 becomes an adsorption site for N_2_ adsorption or that the size of some micropores is reduced because of VB12’s accommodation in larger micropores and nitrogen can access the remaining empty volume of this micropore. Our previous results show that the adsorption–desorption N_2_ isotherms display a hysteresis loop indicating the existence of mesopores of the type IV isotherm characteristic in the presence of narrow slit-like pores according to the IUPAC (International Union of Pure and Applied Chemistry) [29,36]. Adsorption studies have demonstrated that mesopores measuring 3–5 nm and macropores ranging from 10 to 90 nm play a key role in significantly enhancing the adsorption capacity of VB12 [37].

### 2.2. Kinetic Adsorption Modelling Studies

**Figure 2:**
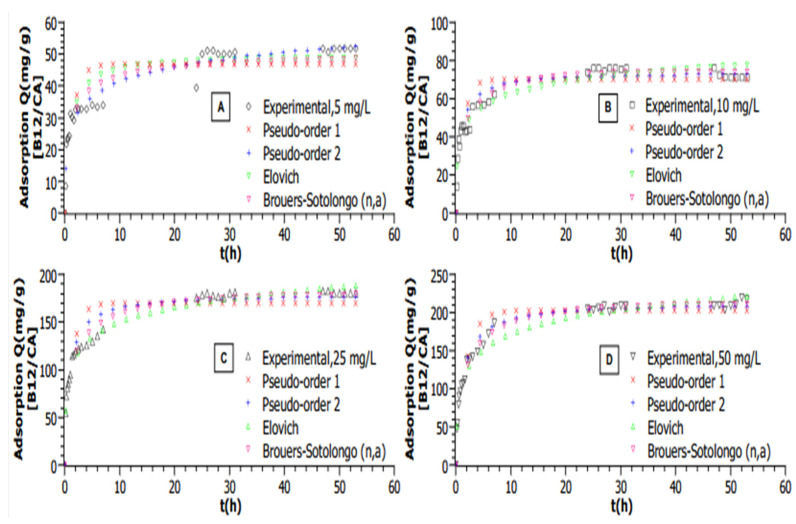
Adsorption kinetic modelling of VB12 adsorption on AC for different initial VB12 concentrations in a volume of 50 mL (T = 25 °C, m_AC_ = 5 mg, pH = 6. (**A**)—[VB12] = 5 mg·L^−1^, (**B**)—[VB12] = 10 mg·L^−1^, (**C**)—[VB12] = 25 mg·L^−1^, (**D**)—[VB12] = 50 mg·L^−1^).

All graphs are plotted with calculated mean values from triplicated experiments; for all experimental data shown in Figure 2, a plateau can be seen, which demonstrates that the equilibrium state has been reached in each case. In this study, multiple adsorption kinetic models were tested to determine the best fit for the adsorption of VB12 on sugarcane-derived activated carbon (AC). The choice of an appropriate model is crucial as it provides insights into the adsorption mechanism, including mass transfer, surface interactions, and potential reaction pathways.

Under most tested conditions, experimental data showed poor alignment with the Pseudo-First Order (PFO) model (Lagergren), which assumes adsorption is governed by physisorption—where the adsorption rate is proportional to the number of available sites. This was evident from higher *AICc* values and significant discrepancies between the experimental and predicted equilibrium capacities. In contrast, the Pseudo-Second Order (PSO) model, which assumes chemisorption as the rate-limiting step, showed a better fit to these data at higher adsorbent doses and elevated temperatures. This model considers adsorption dependent on both surface site availability and adsorbate concentration. The improved fit, indicated by lower *AICc* values and strong correlation coefficients (R^2^ > 0.98), suggests interactions such as valence forces, hydrogen bonding, or charge transfer between VB12 and AC.

The Elovich model, originally developed to describe chemisorption kinetics on heterogeneous surfaces, showed the best fit at the lowest VB12 concentration (5 mg/L). This is because it accounts for the increasing adsorption energy barrier as surface coverage increases, which is relevant in the initial stages of adsorption. However, as equilibrium was approached, this model diverged, making it unsuitable for describing the entire adsorption process. BS model was found to be the most appropriate for describing the adsorption kinetics at nearly all tested conditions. This model incorporates a fractal time component, which accounts for heterogeneity in both adsorption sites and energy distributions. The BS model provided the best statistical fit (lowest AICc and highest R^2^), demonstrating its ability to describe adsorption on structurally complex materials such as AC.

The BS model’s superior fit suggests that adsorption is governed by a combination of surface diffusion and heterogeneous interaction energy rather than a uniform binding mechanism assumed by the Langmuir-based kinetic models. The fractional order of the reaction in the BS model also indicates that the adsorption process does not follow classical reaction kinetics but instead follows a complex, multi-step mechanism.

The adsorption kinetics was investigated as a function of concentration, mass, and temperature.

#### 2.2.1. Influence of Initial VB12 Concentration

In this study, the effect of the initial concentration of VB12 on the adsorption capacity of ACs was investigated for the range 5, 10, 25, and 50 mg·L^−1^. At a constant temperature of 25 °C, as shown in Figure 2, raising the concentration from 5 to 50 mg·L^−1^ allows the ACs to increase their adsorption capacities from 51.8 to 217.5 mg·g^−1^, respectively.

Based on the higher R^2^ values and the lowest *AICc*, the Brouers–Sotolongo (n,a) is the best model (Table 1) for describing kinetic data at almost all concentrations (10 to 50 mg·L^−1^).

Nevertheless, the Elovich model provided the best fit with the lower AICc at a concentration of 5 mg·L^−1^. The phenomenon can be explained by the applicability of the Elovich equation in the initial phase of the adsorption process on a heterogenous surface since the occupation of active sites increased exponentially, whereas, near the equilibrium stage, the curve tends to diverge (when *q*(*t*→∞) = ∞) [38]. The description of adsorption kinetics of the VB12 is better described by the BS (n,a) involving a heterogenous surface with a heterogenous “energy landscape” [39].

#### 2.2.2. Influence of AC Quantity

**Figure 3:**
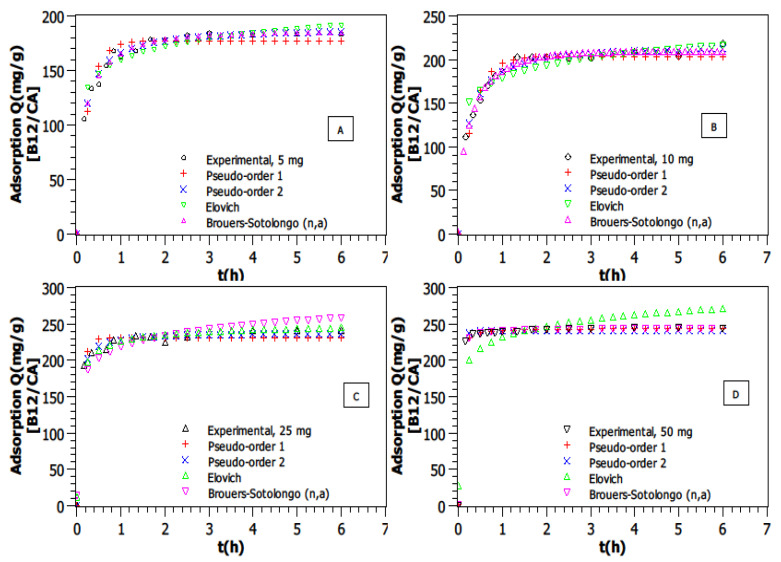
Adsorption kinetic modeling of VB12 for different AC amounts in a volume of 50 mL (T = 25 °C, Ci = 25 mg·L^−1^, pH = 6. (**A**)—mAC = 5 mg, (**B**)—mAC = 10 mg, (**C**)—mAC = 25 mg, (**D**)—mAC = 50 mg).

The effect of AC quantity was investigated at a VB12 concentration of 25 mg·L^−1^_,_ pH = 6, and T = 25 °C. Four masses of AC were used: 5, 10, 25 and 50 mg. The results show that an increase in the AC quantity causes an increase in the amount of adsorbed VB12 (Figure 3). 

Indeed, the adsorption values increased from 184.4 mg·g^−1^ to 245.4 mg·g^−1^ as the AC amount was increased from 5 to 50 mg. This phenomenon can be explained by several factors. As the quantity of AC increases, the total available surface area and the number of active adsorption sites also increase [40]. This provides more binding opportunities for VB12 molecules, leading to higher adsorption capacities [41].

Based on the higher R^2^ values and the lowest *AICc* (in Table 1) for each model and each AC amount, it seems that the Brouers–Sotolongo (n,a) for the low AC mass (5 and 10 mg) and the Pseudo-Second Order model for the rest of the cases are the best models to fit these kinetic adsorption data, as shown in Table 1. Supplementary data associated with this experiment can be found in supplementary information. The BS model performed particularly well at lower adsorbent masses (5 and 10 mg), where surface heterogeneity effects were more pronounced. At higher adsorbent dosages (25 and 50 mg), the PSO model provided a better fit, suggesting that as more adsorption sites become available, the reaction mechanism is closer to a second-order chemisorption process. This result shows a correlation between an increase in the AC amount with the number of unoccupied sites and implies possible chemisorption interaction mechanisms [42].

#### 2.2.3. Influence of Temperature

The adsorption phenomenon was monitored using 5 mg of AC in a VB12 solution of 25 mg·L^−1^ at different temperatures from 25 to 55 °C (Figure S1 in supplementary information). The results indicate that the adsorption capacity increases as the temperature of the system is increased (Figure S1). When the temperature of the solution was raised from 25 °C to 55 °C, the adsorption capacity qe was increased from 184.4 mg·g^−1^ to 297.6 mg·g^−1^. This phenomenon was also described in other publications, suggesting the endothermic adsorption of VB12 on AC and implying that the affinity of the binding sites of adsorbents increases with the temperature [43]. The fact that the adsorption capacity of VB12 on AC increases from 25 to 55 °C indicates that the process is endothermic. As for the thermodynamic analysis, Gibbs free energy change in adsorption (ΔG° (kJ·mol^−1^)), the enthalpy change in adsorption (ΔH° (kJ·mol^−1^)) and the entropy change in adsorption (ΔS° (J·mol^−1^·K^−1^)) were calculated. Based on the experimental temperature range, the negative value of ΔG° (−0.37 kJ·mol^−1^) indicates that the sorption process is thermodynamically favorable and spontaneous. The ΔH° and ΔG° values were calculated from the slope of ln Kd vs. 1/T shown in Table 2. The endothermic nature of the adsorption (as indicated by increasing adsorption capacity with temperature) further supports the relevance of the BS and PSO models over purely physical adsorption models such as PFO.

The diffusion of the adsorbate molecules to the outer and inner boundary layer of the pores of the adsorbent is likely promoted by decreasing the viscosity of the solution and the energy input in the form of molecular agitation in the elevation of temperature.

Based on the higher R^2^ values and the lowest *AICc* (in Table 3) for each model and each AC amount, the Brouers–Sotolongo (n,a) appears to be the best model at 25 and 35 °C while the Pseudo-Second order model provides the best fit at 45 and 35 °C, as shown in Table 3.

#### 2.2.4. Influence of pH

The pH of the VB12 solution has a considerable impact on the state of ionization of the VB12 as well as the surface binding sites of the AC [41]. The role of pH was studied in our recent work [28].

VB12 adsorption on activated carbon is most efficient at pH 6 because of favorable electrochemical and structural interactions.

The pKa value of approximately 3.28 remains predominantly un-ionized at weak acidic conditions, pH = 6, ensuring its structural stability and optimal adsorption behavior [28]. It demonstrates that the quantity of VB12 adsorbed on AC rises when the pH of the solution increases from 2 to 6, reaching a maximum at pH = 6, then decreasing [28]. These results suggest that adsorption of the VB12 on the AC surface affects the protonation of the nitrogen of the α-DMB chain [44].

This promotes efficient binding to the activated carbon’s neutral or slightly negatively charged surface [45]. Docking studies illustrate how VB12′s cobalt ion interacts optimally with the adsorbent’s porous structure, enhancing adsorption through hydrophobic and electrostatic forces.

This can be explained by the weakening of the bond Co-CN, particularly in the pH range from 5.3 to 7.5 [46]. For that reason, in the present study, all experiments were carried out at pH 6, close to the environmental conditions.

The parameters obtained for kinetic modeling of VB12 adsorption on AC for different pH can be found in supplementary information Table S1.

### 2.3. Isotherm Adsorption Modelling Studies

**Figure 4:**
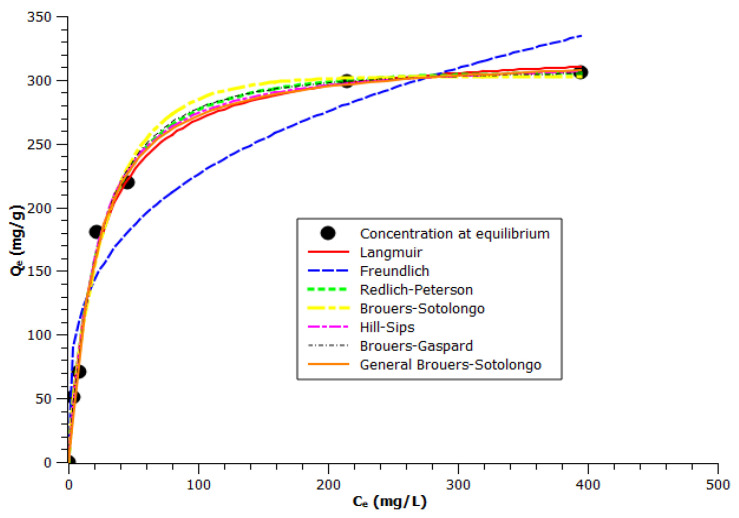
Non-linear isotherm modeling fit related to the adsorption of VB12 onto BagP1.5 at 25 ± 0.1 °C. (A color version of this figure can be viewed online).

Figure 4 shows experimental equilibrium data and the predicted theoretical isotherms for the adsorption of VB12 onto BagP1.5.

A comparison based on Langmuir adsorption capacity *Q°* was made. The results, illustrated in Table 4, show a Langmuir adsorption capacity of about 327.5 mg·g^−1^ at 25 °C, m_AC_ = 5 mg, and pH = 6.

The BG model shows the smallest AIC value, followed by the HS and BS models (Table 3). These models fit more closely to adsorption isotherm data than the classical models generally used, i.e., Freundlich, Langmuir, R-P, or the more complex GBS equation. From Table 4, it was confirmed that equilibrium sorption data were very well represented by the BG isotherm by providing the highest correlation coefficient (*R*^2^ = 0.99) and a low *ARED* (0.6). The BG model, which can be considered as an intermediate between the BS and HS models, provides a better correlation coefficient (*R*^2^ = 0.99) and low *ARED*. Thus, starting from data listed in Table 4, the comparison of tested models for the description of adsorption equilibrium isotherms on sugar cane bagasse AC is as follows: BG > HS > GBS > BS > R-P and Langmuir > Freundlich.

### 2.4. Electrochemical Study of AC and Vitamin B12 Interactions

**Figure 5:**
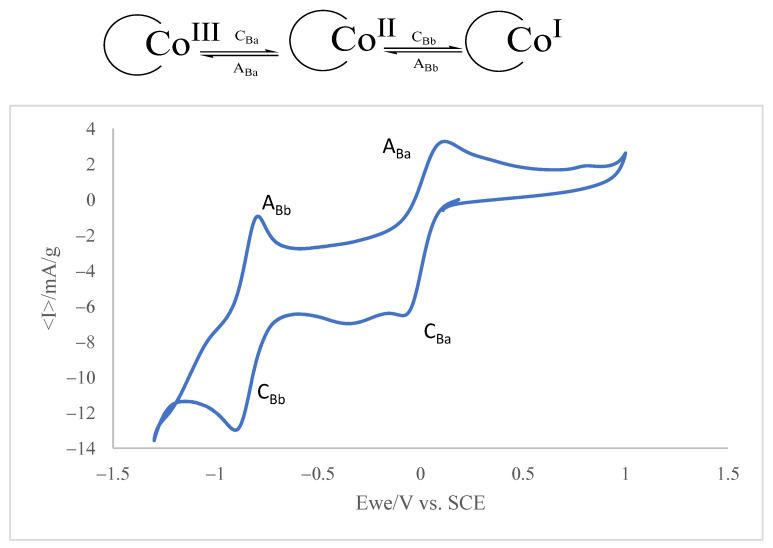
Cyclic voltammogram of a 0.015 mM VB12 solution in air-saturated 0.10 M potassium phosphate aqueous buffer, pH 7.0. Potential scan rate 20 mV·s^−1^ by applying potentials between −1.3 and +1 V. Inset: Scheme of the electrochemical processes involved in VB12 electrochemistry.

To study the interaction of AC with VB12, it is pertinent to explore the voltammetry of both compounds separately. Figure 5 depicts the cyclic voltammogram at the glassy carbon electrode of a VB12 solution in a solution of 0.10 M potassium phosphate aqueous buffer, pH 7.0.

The voltammogram consists of two cathodic peaks at −0.1 (C_Ba_) and −0.9 V vs. Ag/AgCl (C_Bb_) coupled to anodic peaks at 0.05 and −0.8 V, defining two essentially reversible couples. In the solution phase, the voltammetry of the VB12 consists of two essentially reversible one-electron couples corresponding to the Co^III^ to Co^II^ and Co^II^ to Co^I^ reduction processes, respectively. This response is in agreement with literature data [47,48] and can be described in terms of two successive one-electron transfers corresponding to the Co(III)/Co(II) and Co(II)/Co(I) couples (see scheme in the inset in Figure 5).

In the case of AC no representative signals were registered compared with the potassium phosphate aqueous buffer.

An additional series of experiments were carried out on a phosphate buffer solution of VB12 also containing a suspension of AC (ca. 1 mg·mL^−1^). At the beginning (time = 0 min) of the experiment, the voltammogram shows the two signals characteristic of VB12 such as in Figure 5. In the presence of activated carbon, a significant fraction of the VB12 becomes adsorbed, and then a reduction in species in solution is accompanied by a reduction in the adsorbed species. This takes place at slightly less positive potentials. As a result, the voltammogram shows peak splitting. However, after overnight incubation (12 h) under stirring, the voltammograms show significant changes. As can be seen in Figure 5, the intensity of the C_Ba_/A_Ba_ and C_Bb_/A_Bb_ couples decreases and exhibits peak splitting, which is particularly relevant in the C_Ba_/A_Ba_ couple.

**Figure 6:**
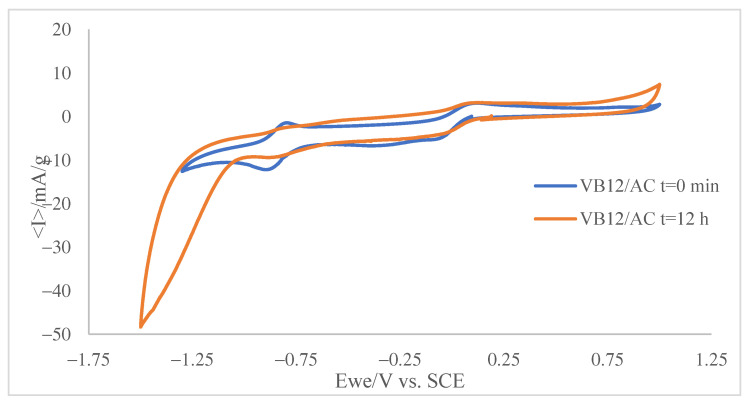
Superposition between the cyclic voltammogram recorded at GCE on 0.015 mM VB12 solution in air-saturated 0.10 M potassium phosphate aqueous buffer, pH 7.0 in the presence of suspended (1 mg·mL^−1^) AC after overnight incubation (12 h) under stirring. Voltammograms recorded at t = 12 min (green) and t = 0 min (red) of stirring. Potential scan rate 20 mV·s^−1^.

The observed peak splitting, attributable to the superposition of the voltammetric responses of ‘free’ VB12 and VB12 adsorbed on AC, is consistent with molecular modeling predictions, as the voltammograms in Figure 6 indicate that the adsorbed VB12 is reduced at potentials slightly less negative than the ‘free’ VB12 in solution.

### 2.5. Molecular Modelling

To better interpret the results at the molecular level, molecular modeling was applied. As three-dimensional models of activated carbon are difficult to generate, we had to rely on the unique possible option, the work presented by Huang and coworkers [49]. This model provides 18 non-redundant cuboids of the AC models [49] with whom VB12 could potentially interact. To ascertain which are the best possible interaction sites and orientations, the three-dimensional builder GaudiMM was used to dock VB12 into each cuboid of the porous material. From the four replicas performed for each cuboid, all best solutions show no poor contacts (clashes) between the VB12 molecule and the AC structure, implying that the VB12 can locate hosting cavities with great matching (Table 5).

**Figure 7:**
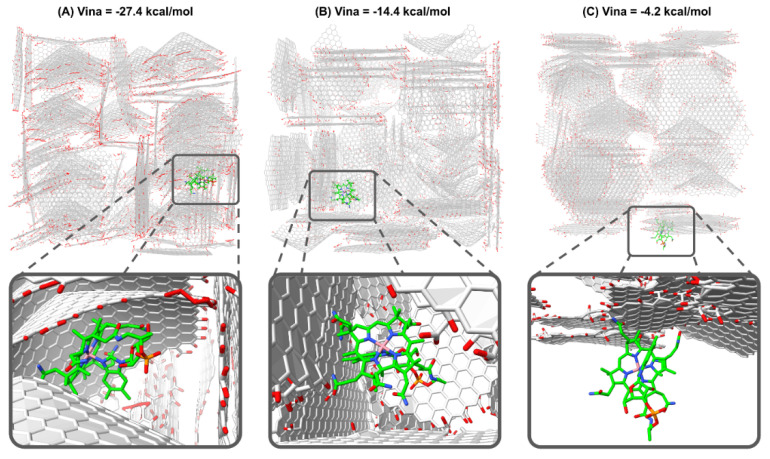
Docking solutions for the VB12 molecule bound to AC structure. VB12 is depicted in green sticks, while AC is shown in grey, otherwise standard representation of atoms are used: red for oxygen, blue for nitrogen, different degrees of gray for carbon, orange for phosphorous and pink for cobalt. (**A**) Vina score = −27.4 kcal·mol^−1^, VB12 is bound near a conic sheet of the AC structure. (**B**) Vina score = −14.4 kcal·mol^−1^, VB12 is bound near several plain sheets of the AC structure. (**C**) Vina score = −4.2 kcal·mol^−1^, VB12 is bound at the exterior of the AC structure.

Similarly to protein–ligand docking in biochemistry, here GaudiMM serves to explore for each cuboid which are the most favorable regions and orientations for VB12. Comparison with the whole set of calculations for all the replicates and cuboids allows us to determine the most probable kind of interactions and specificities in the VB12-AC complementarities including the shape of the carbon layers. When examining GaudiMM docking results under the scope of the vina scoring function, computations demonstrate a range in binding energy from −1 to −27.4 kcal·mol^−1^ (Table 5). The most stable interactions are observed in model AC1, as it shows the best score in cuboid AC1.2 (−27.4 kcal·mol^−1^). Visual inspection of all the complexes was used to examine the cause of this stability further. It appears that in the best energy complex, VB12 is well integrated into a cavity with a conic form and that this tendency is systematically observed for a large number of docking solutions with good vina scores (lower than −16.0 kcal·mol^−1^, Figure 7A).

For cases with worse predicted binding energies, docking poses with intermediate vina score (between −12.0 and −6.0 kcal·mol^−1^, Figure 7B) present VB12 mostly interacting with one or more plain sheet/s of the AC., while in the worst scored complexes (higher than −12.0 kcal·mol^−1^, Figure 7C) VB12 is usually found at the exterior surface of the cuboid.

The calculations imply that places near curved sheets are preferable for VB12 binding into the activated carbon superstructure. This preferential affinity is owing to strong hydrophobic contacts between the sheet and a major percentage of VB12 which also contemplate a better shape complementarity. Despite the substantial packing effect between the AC sheets and the corrin part of VB12, it is striking that those solutions show one side of the macrocycle to be exposed to the cavity space, which would allow it to catalyze reactions with entering substrates. Similarly, attaching VB12 molecules to these sites would improve the accessibility of metal sites for electron exchange, changing the reduction potentials to less negative values.

The low energy calculated VB12-AC complexes (vina score > −16.0 kcal·mol^−1^) highlight that hydrophobic interactions together with shape complementarity are the most important driving forces for molecular recognition; a type of interactions also present in other highly dimensional systems such as protein interactions with small molecules [50,51]. Such observation is in agreement with the explanation given by some authors since the distance from curved hard walls is close to the distance of the molecule (VB12 around 2.06 nm) [44,52].

The modeling exercise presented in this study considers the best model available to date for AC structure.

It could be argued that the AC model has a series of limitations that include the absence of modeling capillary condensation of VB12 in the pores with H_2_O and interacts with one or more plain sheet/s of the mesoporous BagP1.5 sample [53,54] or that numerous abnormalities, impurities, and imperfections in graphite sheets might disrupt adsorption with varying pore geometries at the external surface [55,56]. In this case, hydrogen bonds can be formed with wall AC and the oxygen atom on P=O, the OH group, and NH_2_ (corrin cycle) of the VB12. However, to the best of our knowledge, the approach used here is the only accessible one that already has some evidence for molecular interaction and sustains future studies, including the design of VB12 derivatives.

## 3. Discussion

The Pseudo-First Order and the empirical Elovich equations, which are generally used to fit adsorption kinetic data in aqueous environmental systems, provide poor fitting of experimental adsorption kinetic data of VB12 on the AC with a large difference between the experiment (qe exp) and the calculation (qe calc) and low linear regression correlation coefficients R^2^. Particularly, the empirical Elovich equation, which has a wrong asymptotic limit, varying as log (t), provides low R^2^ values and cannot give a reliable description of the whole kinetics. In some cases, the Pseudo-Second Order model is able to fit these data with good accuracy, assuming a chemical sorption mechanism that may involve chemical interactions or exchange of electrons between VB12 with carboxylic groups at the AC surface [11].

Simple theoretical considerations based on some fundamental theories (i.e., adsorption on gases [57]) suggest that model approximations fit very well despite offering no insights into the mechanistic aspects [58]. The behaviors predicted by many different theoretical approaches do not reflect the reality of the problem. Sorbing materials such as AC derived from natural precursors induce structural and energetic heterogeneities, which are better described by the BS (n,a) kinetic model [17,59].

BS (*n*,*a*) is shown to be generally the best model for fitting most adsorption kinetic data due to the fact that this Weibull formula can assign natural limits to the true kinetics and the quantities characterizing the kinetics, i.e., the value of the maximum sorption q_e_, the fractal time exponent α, can be calculated. This result indicates that a real reaction order is difficult to calculate because of the complex adsorption mechanism taking place on a heterogeneous surface such as that of the AC. This assumption is verified by the isotherm adsorption data modeling, showing that Langmuir and GBS models are not suitable for predicting these adsorption isotherm data. The Weibull formula of the GBS isotherm that is generally used for pharmacokinetics systems [22] is generally the model used for describing the adsorption and desorption isotherms. Such a fitting tendency would refer to the presence of active sites with heterogeneous sorption interactions [17] in agreement with previous works [60]. In this case, model fits are ranked according to their AICc values; the BG model is the best model with the lowest AICc value, followed by HS. The AICc penalizes models that have a lot of free parameters more strongly.

The use of an intermediate value for n = 1.5 gives an intermediate equation between the HS and the Weibull models, which is the BG kinetics with statistical quantities being intermediate between the ones obtained from HS and Weibull formulas. This proposed intermediate kinetics for n = 1.5. BG gives as well a good representation of the real sorption isotherms such as benzene [26]. Some publications introduced BG isotherm as the best to describe the adsorption of two common dyes, methylene blue (MB) and methyl orange (MO), onto a heterogeneous AC surface and show “a” and “b” parameters are clearly correlated with pH and temperature [61,62].

In our case, parameter “a” is close to 1, which may indicate one molecule adsorbed by an active site and an equilibrium between the adsorption and desorption processes, parameter b is linked to equilibrium concentration, and parameter “c” is fixed to 0.5 related to the agglomeration and clustering of AC particles, or the fractal distribution of mesopores [16,26]. The voltammetric study reinforces the assumption that in the presence of activated carbon, a significant fraction of VB12 becomes adsorbed, and then a reduction in the species in solution is accompanied by a reduction in the adsorbed species. This takes place at slightly less positive potentials and shows peak splitting that can be associated with the superposition of the voltammetric responses of ‘free’ VB12 and VB12 adsorbed on AC.

Such a tendency would most likely provide insight into the energy heterogeneity at the AC surface, which is caused by its geometric and chemical properties. Geometric heterogeneity results from variances in the size and form of pores, fractures, and pits. Chemical heterogeneity is connected with several functional groups, such as carboxyl surface groups, hydroxyl groups, ethers, and the aromatic skeleton. These groups may cause electrostatic attraction, complexation, or hydrophobic interactions with the solute molecule. Computational data allow us to calculate low-energy VB12-AC complexes, indicating that hydrophobic interactions, together with shape complementarity, are the most important driving forces of the interaction of the VB12 with the AC surface.

Hydrophobicity is due to water molecules that do not form hydrogen bonds with carbon surfaces and expose a surface without functional groups. In this case, VB12 absorption occurs in a capillary condensation [54,63,64]. Molecular modeling shows variation in binding energy, and most stable interactions with VB12 were made in a cavity with a conic form that would not exclude hydrogen bond formation with AC wall AC and the oxygen atom on P=O, the OH group, and NH_2_ (corrin cycle) of the VB12.

Thus, such trends would lead to a heterogeneous sorption energy landscape, explaining, therefore, the good fit of the Brouers–Sotolongo kinetic model. This result may suggest monolayer adsorption onto a heterogeneous surface due to the impossibility of obtaining multilayer adsorption because of VB12 size.

## 4. Experimental Section

### 4.1. Carbon Materials’ Preparation

The AC was made from sugarcane bagasse. This biomass is a sugarcane industry waste by-product, abundantly available at a minimal price, and is known as an excellent precursor for the development of AC with different textural properties [65].

The sugarcane bagasse came from the Gardel manufacturing company in Moule, Guadeloupe, French West Indies. Bagasse has been sun-dried before being dried in an oven at 105 °C for 48 h to eliminate moisture. The material was then ground and sieved [66]. The fraction containing particles ranging in size from 0.4 to 1 mm was employed. Bagasse was subjected to a chemical activation: approximately 3 g of the precursor were placed in a 100 mL beaker, impregnated with phosphoric acid (H_3_PO_4_) at 85% for 24 h, in order to facilitate access of the acid inside the particles [67].

The impregnation proportion was 1.5:1 (g H_3_PO_4_/g precursor), giving samples BagP1.5. After impregnation, the samples were dried in an oven at 110 °C for 4 h before being placed in a horizontal furnace (Thermolyne^®^ F 21100) for pyrolysis with a nitrogen flow of 80 mL·min^−1^.

VB12 was obtained from SIGMA-ALDRICH. A stock solution of cobalamin was made by dissolving VB12 in demineralized water to a concentration of 50 mg·L^−1^. Dilutions were made one after the other to obtain other concentrations.

VB12 solutions were then diluted in distilled water to obtain a concentration of 25 mg·L^−1^. Adsorption kinetics of VB12 onto the AC were conducted at four VB12 (5, 10, 25, and 50 mg·L^−1^) in a volume of 50 mL.

The adsorption kinetics of VB12 were conducted using amber bottles to reduce light exposure, however, recent studies have demonstrated that VB12 exhibits sufficient photostability under typical conditions, indicating that light protection might not be strictly necessary [27,37,68]. The samples were kept in a heated bath at 25 ± 0.1 °C and agitated at 300 rpm.

Aliquots of the samples were collected using a syringe and a 26 mm diameter Minisart NML cellulose acetate filter with a pore diameter Ø ≤ 5 µm. Measurements were collected every 10 min during the first hour of the reaction, every 20 min during the second hour, every 30 min during the third hour, and finally every hour until equilibrium was reached [25]. Every experiment was conducted twice, and the mean values were documented. It was discovered that the standard deviation was ± 4.4%.

VB12 concentration was assessed using a UV-visible spectrophotometer (SECOMAM, UV UviLine 9400) at 362 nm [37]. The pH was adjusted with a pH meter (Meterlab^®^ PHM 220). The quantity of VB12 absorbed onto the AC at any given time was calculated using the concentration in the solution prior to and after adsorption. At each current time, the amount of adsorbed VB12 onto AC, *q_t_* (mg·g^−1^), was given by the mass balance equation as follows:

(1) $$q_{t}=\frac{V\left(C_{0}-C_{e}\right)}{M}$$

where *C*_0_ and *Ce* are VB12′s initial and equilibrium liquid phase concentrations (mg·L^−1^), respectively, *V* is the solution volume (L), and *M* is the adsorbent mass (g).

In this study, the theoretically predicted modeling data were calculated using the Levenberg–Marquardt normalized iteration algorithm included in SciDAVis^®^ statistics software version 0.2.4 (fourth update in the 0.2 series) for non-linear assessment. The parameters estimated through linearization were used as initial values for the iterative processes related to non-linear regression [17,39].

### 4.2. Specific Surface Area and Pore Size Determination

Details of the BET theory (Brunauer, Emmett, and Teller) analysis process, pore size distribution was evaluated by using the Barrett–Joyner–Halenda (BJH) model, micropore volume using t-Plot micropore volume of the BagP1.5 and BagP1.5@VB12 was performed as described elsewhere [28].

### 4.3. Isotherm Adsorption Studies

Adsorption isotherms of VB12 on the AC were determined in a volume of 50 mL at the following concentrations of VB12 in water: 5, 10, 25, 50, 100, 150, 200, 250, 500 mg·L^−1^.

The samples were immersed in a heated water bath at 25 ± 0.1 °C and stirred at 300 rpm.

### 4.4. Adsorption Kinetic Data Modelling

In a previous investigation, it was shown that a fractal kinetic can also be a useful theoretical tool for studying adsorption processes and describing the dynamics of adsorption phenomena on AC [69]. An equation was developed from the general fractional differential equation [13,14]:

(2) $$-\frac{dq\left(t\right)}{dt^{\alpha }}=k_{\alpha ,n}q^{n}\left(t\right)$$

$q\left(t\right)$ is the mass of solute adsorbed per gram of AC;$\;q_{e}$, is the maximum adsorbed quantity, *n* is the reaction order, and *α* is a fractional time index. When *n* and *α* are different from 1, $t^{\alpha }$ is the time-independent rate constant, and the relevant quantity characterizing the time evolution of the process is the characteristic time $\tau_{\alpha }$_._

The Elovich model is a rate equation initially suggested by Roginsky and Zeldovich in 1934 to explain the kinetics of the adsorption of carbon monoxide on manganese dioxide [70]. This model relies on a kinetic principle that predicts that adsorption sites expand exponentially with adsorption, and it might imply multilayer adsorption [71]. Chien and Clayton simplified the Elovich equation by assuming αβt > 1 and applying boundary conditions q(t = 0) = 0, resulting in [72]:

(3) $$q_{t}=\ln\frac{\alpha \beta }{\beta }+\frac{1}{\beta }\ln t$$

where *α* is the initial adsorption rate (mg·g^−1^·min^−1^) and *β* is the desorption constant (g·mg^−1^) during the experiment [17].

The Brouers–Sotolongo (BS (*n*,*α*)) generalized fractal kinetic equation is given when *α* ≠ 1 and *n* = 1, obtaining the Weibull kinetics or Avrami kinetics from the theory of crystallization [73], expressed in the following equation in a more usual form:

(4) $$q_{t}=q_{m}({1-\left(1+\left(n-1\right)\times {\left(\frac{t}{\tau_{\alpha }}\right)}^{\alpha }\right)}^{-\frac{1}{n-1}}$$

where *q_t_* and *q_m_* are the time-dependent and maximum adsorbed quantities, in (mg·g^−1^); n is the fractional reaction order parameter of the reaction; α is the fractal coefficient expressing macroscopically the complexity of the sorbent–sorbate couple, expressing memory effects because of the heterogeneity of the sorption free energies and the resulting distribution of reaction constants given by the Arrhenius law, and $\tau_{\alpha }$ is a characteristic time (min) [22,41].

### 4.5. Adsorption Isotherm Data Modelling

Isotherm models give some insight into the sorption mechanism as well as the solute’s affinity for the surface adsorbent. The following equation represents the General Brouers–Sotolongo (GBS) isotherm [22]:

(5) $$q_{e}=q_{m}\left[1-{\left(1+c{\left(\frac{x}{b}\right)}^{a}\right)}^{-\frac{1}{c}}\right]$$

where *q_e_* is the adsorbed quantity at equilibrium (mg·g^−1^), *q_m_* is the maximum adsorbed quantity (mg·g^−1^), x is the residual solute concentration in solution at equilibrium (mg·L^−1^), *b* is the isotherm constant (mg·L^−1^), *a* is a coefficient dependent on the fractal nature of the system and this exponent is a measure of the width of the adsorption energy distribution and energy heterogeneity of the sorbent surface [74,75], and *c* is the coefficient related to its cluster organization [76].

If *c* = 1 and *a* = 1, GBS reduces to the Langmuir model being the most often used isotherm model for the sorption of a solute from a liquid solution (Equation (5)). This model is based on the assumption that sorption sites are identical and energetically equivalent and that the solute is immobilized under the form of monolayer coverage [57,58].

(6) qe=qmxb1+xb 

The Langmuir isotherm can be written in a more familiar form:

(7) $$q_{e}=\frac{Q^{{}^{\circ}}\left(K_{L}C_{e}\right)}{1+K_{L}C_{e}}$$

where *Q*° is the Langmuir monolayer adsorption capacity (mg·g^−1^) and *K_L_*_,_ the Langmuir isotherm constant (L·mg^−1^);

The Hill–Sips (HS) isotherm model can be derived from GBS when *c* = 1 (Equation (5)). HS isotherm model is a combined form of Langmuir and Freundlich expressions deduced for predicting the heterogeneous adsorption systems and the limitation of the rising adsorbate concentration associated with the Freundlich isotherm model [76]. At high adsorbate concentration, it predicts monolayer adsorption characteristics of Langmuir, while at low adsorbate concentration, it reduces to Freundlich isotherm [77].

(8) qe=qm xba1+ xba

The mathematical equations of Freundlich isotherm is described by Equation (9):

(9) $$q_{e}={K_{L}\left(C_{e}\right)}^{\frac{1}{n_{F}}}\;$$

where *K_F_* is the Freundlich isotherm constant [(mg·g^−1^)(L·mg^−1^)((1 − n/)n)]; n_F_ = 1/n, the Freundlich exponent (dimensionless);

For *c* = 0, Equation (5) recovers the normal Brouers–Sotolongo (BS) isotherm equation (Equation (10)). BS is basically a deformed Weibull exponential equation that was developed to describe a complex adsorption process involving highly heterogeneous systems. This model, which considers a pattern of sorption energy distribution, is suitable for describing adsorption phenomena involving sorbing materials, even if they exhibit different chemical and structural characteristics [60]. The surface of the sorbing material is assumed to contain a finite number of patches of sites of different sorption energies [16].

(10) $$q_{e}={q_{m}\left(1-\exp\left(-\frac{x}{b}\right)\right)}^{a})$$

In many cases, it was shown that the choice between HS and BS isotherms is difficult. It is thus proposed to use an intermediate value for *c* (*c* = ½) and introduce a new intermediate formula, which is the BG isotherm equation [75]:

(11) qe=qmxba 1+0.25 xba1+0.5 xba)

All these equations have good physical asymptotic behaviors:

As $\frac{q_{e}}{q_{m}}\to 1$ for *x* >> b

and

qeqm→(xb)a for *x* << b

Redlich–Peterson (R-P) isotherm model that incorporates the features of the Langmuir and Freundlich models into a single equation and presents a general isotherm [75] is also commonly used. This model can be applied to describe the adsorption process at a high solute concentration [76].

(12) $$q_{e}=\frac{A_{R-P}C_{e}}{1+K_{R-P}({C_{e})}^{\beta }}$$

where *A_R-P_* is the Redlich–Peterson isotherm constant [(L·mg^−1^)*β*]; *K*_R-P_ is the Redlich–Peterson isotherm constant (L·g^−1^); β: Redlich–Peterson exponent (dimensionless);

This equation does not have the physical correct asymptotic limit since it gives:

$q_{e}\to ABx$ for *x* << *B*

And

$q_{e}\to 0$ (if *a* > 1) or $q_{e}\to +\infty$ for x >> *B*

### 4.6. Statistical Tools Used to Determine the Best Fitting Model

Statistical tests were employed to determine the goodness of fit of the analyzed models. The correlation coefficient R^2^ is not a reliable criterion for model selection [17,38].

The average relative error deviation (*ARED*) is an error function relying on the minimization of the fractional error distribution throughout the whole concentration range [17]. If the percentage deviation error was less than 10%, the modeling results were deemed adequate for accurately describing the adsorption process.

(13) $$ARED=\frac{1}{N}\sum \left|\frac{q_{e,exp}-q_{e,cal}}{q_{e,cal}}\right|*100\;$$

where *N* is the number of experimental data points, *q_e,cal_* (mg·g^−1^) is the theoretically calculated adsorption capacity at equilibrium, and *q_e,exp_* (mg·g^−1^) is the experimental adsorption capacity at equilibrium.

On the other side, the Akaike information criterion (*AIC*) measures a statistical model’s quality of fit. This criteria proposed by Akaike (1973) stems from information theory and is based on Kullback and Leibler (1951) is a measure (a “distance” in a heuristic sense) between conceptual reality and the approximation model specified for continuous functions as the integral [78,79,80,81,82,83]. It is the introduction of a new model selection criterion that penalizes models for which the explained variable does not provide sufficient information and selects the best model with the smallest Kullback–Leibler distance, given these data and candidate models. Models with too few parameters have a bias, whereas models with too many parameters may have poor precision [81].

(14) $$AIC=n\cdot \ln\left(\frac{SSR}{n}\right)+2k$$

where k is the number of parameters in the model, and *n* is the number of data points.

The model with the minimal *AIC* value of all candidate models indicates the best model [84]. When *n* is small compared to *k* (*n/k* < 40), the corrected *AIC* value (*AICc*) is more accurate [85]:

(15) $$AICc=AIC+\left(\frac{2k\;\cdot \left(k+1\right)}{n-k-1}\right)$$

This concept helps to reduce model selection bias effects and avoids loss of information due to discrimination of models which are almost equally probable [84,85].

### 4.7. Electrochemical Experiments

The main objective was to be treated using solutions in a biological medium (phosphate buffer at pH 7.0) by means of conventional solution phase electrochemistry in view of the high hydrosolubility of VB12. This methodology was previously used for different bioelectrochemical studies [59,60,61]. Electrochemical experiments were conducted at 298 ± 1K in a thermostated cell using CH I660 equipment. A typical three-electrode arrangement was used: a BAS MF2012 glassy carbon working electrode (GCE) (geometrical area 0.071 cm^2^), a platinum wire auxiliary electrode, and an Ag/AgCl (3 M NaCl) reference electrode. VB12 from Sigma-Aldrich and AC material from Sigma-Aldrich were used as received. Voltammetric measurements were performed in a three-electrode cell using a CH I420 device (Cambria Scientific, Llwynhendy, Llanelli, UK).

For the study, 20 mg (0.015 mM) of VB12 was dissolved in 5 mL of phosphate buffer, performing the first set of voltammetric experiments. After that, 60 mg of BagP1.5 (3 equiv. *w*/*w*) was added to the solution, performing the second set of voltammetric experiments. Finally, the mixture was stirred overnight, and the third set of voltammetric experiments was conducted.

### 4.8. Molecular Modeling

As a last part of our study, we applied a molecular modeling protocol to obtain three-dimensional models of the interaction between VB12 and AC. First, the atomic structure of VB12 was obtained from the structure available in the Protein Data Bank (PDB code 1ddy). Subsequently, this structure has been docked into the AC atomistic models proposed by Cannon et al. [49]. Cannon suggested three AC structures of size 200 × 200 × 200 Å (here denominated cubes AC1, AC2, and AC3) that contain a different percentage of sheets with curvature (70%, 50%, and 30%, respectively) and fit the experimental characterizations of the element composition and porosity of the AC. Each model consists of eight smaller cuboids measuring 100 × 100 × 100 Å. During the dockings, all non-redundant “small” cuboids (eight for AC1, five for AC2, and five for AC3) were sampled.

At the start of this study, two approaches were proposed: screening cuboids for cavities larger than VB12 (1200 Å^3^) and docking in the best sites and blind docking of the ligand directly in the entire cuboid. For volume calculations, the surfnet algorithm (SURFNET: a program for visualizing molecular surfaces, cavities, and intermolecular interactions [86]. As implemented in UCSF, Chimera was used with a cut-off distance of 6 Å and a proof size of 18 Å^3^ [87]. Regarding dockings, they were performed with the GaudiMM platform [88]. GaudiMM is a multi-objective genetic algorithm with a wide range of applications. Calculations were carried out allowing full flexibility to the VB12 torsional angles and using two objectives to evaluate its binding within the AC structure: clashes in order to minimize bad contacts of the VB12 molecule with the overall skeleton of AC and Vina score to obtain an approximated energetic value [89]. The .yaml input file used in the GaudiMM calculations is provided in the supporting information.

Test calculations of both procedures were performed on cuboid AC1.1 (selected randomly). As similar results were obtained with both approaches, the blind docking protocol has been selected for the rest of the study as calculations carried out with this approach resulted globally less demanding in computational resources and faster analysis. Because the conformational space to cover is extremely large, four independent replicas were performed for each of the 18 cuboids.

## 5. Conclusions

The adsorption of VB12 onto sugarcane bagasse-derived AC was investigated to develop a hybrid material of interest to solve the serious environmental problem of water and soil contamination by chlorinated pesticides such as CLD.

This study clearly demonstrates that it is possible to form a hybrid material by chemisorption of VB12 onto activated carbon. This material is characterized by the inclusion of VB12 inside the large micropores and mesopores of the activated carbon, as evidenced by comparing the pore distribution of the carbon material before and after VB12 adsorption. These results are supported by molecular modeling studies showing that VB12 is included within the cavities of the carbon material, which have a conical shape, allowing interactions between VB12 and the pore walls, thereby stabilizing the molecule inside the pore. These interactions likely lead to electron transfer between the surface groups of the activated carbon and cobalamin, resulting in modification of the cobalamin’s electrochemical features.

## Figures and Tables

**Table 1:** Parameters obtained for kinetic modeling of VB12 adsorption on AC for different initial concentrations (T = 25 °C, mAC = 5 mg, pH = 6, V = 50 mL).

		Initial Concentration VB12			
**Model**	**Parameters**	**5 mg·L^−1^**	**10 mg·L^−1^**	**25 mg·L^−1^**	**50 mg·L^−1^**
Pseudo-First Order	*qe_exp_*	51.8	76.1	182.85	217.5
	*qe_calc_*	46.9	70.4	169.8	202.4
	*k*_1_	0.7	0.8	0.8	0.6
	*ARED*	2.6	3.1	3.6	5.0
	*AICc*	119.34	129.73	183.88	175.35
	*R* ^2^	0.79	0.86	0.87	0.94
Pseudo-Second Order	*qe_exp_*	51.8	76.1	182.85	217.5
	*qe_calc_*	49.8	73.9	179.1	212.9
	*k*_2_	0.02	0.02	0.006	0.004
	*ARED*	1.1	0.9	2.0	2.1
	*AICc*	99.9	104.2	154.5	138.2
	*R* ^2^	0.89	0.94	0.95	0.98
Elovich	*qe_exp_*	51.8	76.1	182.85	217.5
	*qe_calc_*	52.9	74.1	185.2	217.4
	*α*	328.6	784.6	1748.7	1024.7
	*β*	0.2	0.1	0.04	0.03
	*ARED*	1.5	2.0	0.2	0.9
	*AICc*	70.7	99.0	105.9	141.8
	*R* ^2^	0.95	0.93	0.98	0.97
BS (*n*,*a*)	*qe_exp_*	51.8	76.1	182.85	217.5
	*qe_calc_*	51.1	73.9	184.5	214.2
	*n*	2.0	0.7	0.5	1.1
	*τ*	0.9	2.6	4.39	2.1
	*α*	0.7	0.4	0.3	0.6
	*ARED*	25.5	1.5	0.2	0.2
	*AICc*	146.8	87.3	94.2	110.7
	*R* ^2^	0.92	0.96	0.99	0.99

**Table 2:** Thermodynamic parameters for VB12 adsorption on AC for different temperatures (mAC = 5 mg, Ci = 25 mg·L^−1^, pH = 6, V = 50 mL).

	∆G°(kJ·mol^−1^)				∆H° (kJ·mol^−1^)	∆S° (J·mol^−1^·K^−1^)
T °C	25 °C	35 °C	45 °C	55 °C		
	−0.37	−0.79	−0.65	−0.80	3.64	13.67

**Table 3:** Parameters obtained for kinetic modeling of VB12 adsorption on AC for different temperatures (mAC = 5 mg, Ci = 25 mg·L^−1^, pH = 6, V = 50 mL).

		T°C			
Model	Parameters	25 °C	35 °C	45 °C	55 °C
Pseudo-First Order	*qe_exp_*	184.4	219.2	234.8	297.6
	*qe_calc_*	169.8	193.01	218.3	268.9
	*k*_1_	0.8	0.858	0.70	0.49
	*ARED*	6690.0	2.10	4.40	4.11
	*AICc*	186.2	131.2	194.8	179.2
	*R* ^2^	0.87	0.94	0.86	0.94
Pseudo-Second Order	*qe_exp_*	184.4	219.2	234.8	297.61
	*qe_calc_*	179.1	203.9	228.9	285.12
	*k*_2_	0.01	0.0063	0.0050	0.0026
	*ARED*	3927.2	0.21	2.09	0.08
	*AICc*	154.5	−86.6	136.0	−90.0
	*R* ^2^	0.95	0.98	0.94	0.98
Elovich	*qe_exp_*	184.4	219.2	234.78	297.61
	*qe_calc_*	185.2	207.56	230.60	277.29
	*α*	1748.7	13,565.40	16,699.95	6885.41
	*β*	0.04	0.05	0.05	0.03
	*ARED*	755.3	7.88	5.55	17.47
	*AICc*	105.9	170.82	199.56	226.47
	*R* ^2^	0.98	0.89	0.90	0.83
BS (n,α)	*qe_exp_*	184.4	219.2	234.8	297.6
	*qe_calc_*	184.5	257.5	229.2	315.3
	*n*	0.90	18.24	0.70	2.99
	*τ*	0.25	0.18	2.62	1.13
	*α*	0.52	4.51	0.38	1.00
	*ARED*	0.11	0.32	0.28	1.32
	*AICc*	−61.1	19.65	−46.35	−38.3
	*R* ^2^	0.99	0.99	0.98	0.98

**Table 4:** Isotherm modeling parameters related to the adsorption of VB12 onto BagP1.5: non-linear approach (mAC = 5 mg, V = 50 mL at T = 25 °C and time at equilibrium te = 48 h).

		Parameters								
		qe exp	qe calc	Constants				ARED	AICc	R^2^
model	Langmuir	306.3	305.7		*Q°* = 327.5			127.6	85.8	0.99
	Freundlich	306.3	334.3	*K_F_* = 60.5	*n* = 3.5			14.0	29.6	0.92
	R-P	306.3	305.3	*A_R-P_* = 13.3	*A_R-P_* = 0.03	*β* = 1.0		1.4	30.6	0.99
	BS	306.3	302.4	*q_m_* = 302.6	*K_w_* = 0.05	α = 0.9		0.8	11.4	0.99
	HS	306.3	303.4	*q_m_* = 316.4	*a* = 1.1	*b* = 19.9		0.1	0.7	0.99
	BG	306.3	303.1	*q_m_* = 308.2	*a* = 1.0	*b* = 23.5		0.6	−6.7	0.99
	GBS	306.3	307.4	*q_m_* = 322.6	*a* = 1.2	*b* = 18.2	C = 1.3	0.4	8.0	0.99

**Table 5:** Clashes (Å^3^) and vina (kcal·mol^−1^) scores for the best structure obtained in each calculation.

	Replica 01		Replica 02		Replica 03		Replica 04	
Cuboid	Clashes	Vina	Clashes	Vina	Clashes	Vina	Clashes	Vina
Cube AC1								
AC1.1	0	−18.869	0	−12.627	0	−21.856	0	−11.773
AC1.2	0	−11.925	0	−18.394	0	−9.264	0	−27.395
AC1.3	0	−21.889	0	−12.380	0	−10.253	0	−10.175
AC1.4	0	−8.811	0	−10.261	0	−10.967	0	−12.834
AC1.5	0	−7.958	0	−8.568	0	−9.725	0	−0.997
AC1.6	0	−12.325	0	−12.507	0	−10.786	0	−13.311
AC1.7	0	−14.519	0	−10.569	0	−12.877	0	−8.443
AC1.8	0	−12.557	0	−13.934	0	−12.922	0	−10.059
Cube AC2								
AC2.1	1.661	−11.776	0	−8.624	0	−23.528	0	−14.500
AC2.2	0	−9.162	0	−15.800	0	−15.330	0	−9.861
AC2.3	0	−12.602	0	−4.211	0	−13.029	0	−10.548
AC2.4	0	−16.726	0	−11.247	0	−10.704	0	−16.304
AC2.5	0	−9.659	0	−14.604	0	−14.403	0	−14.770
Cube AC3								
AC3.1	0	−10.438	0	−19.253	0	−16.519	0	−8.455
AC3.2	0	−11.276	0	−17.098	0	−5.156	0	−15.194
AC3.3	0	−15.103	0	−15.553	0	−19.563	0	−15.175
AC3.4	0	−11.071	0	−10.043	0	−9.269	0	−9.113
AC3.5	0	−10.731	0	−8.743	0	−14.941	0	−14.440

## Data Availability

Data is unavailable due to privacy.

## Supplementary Materials

The following supporting information can be downloaded at: https://www.mdpi.com/article/10.3390/molecules30102096/s1, Figure S1: Maximum adsorption capacity obtained for VB12 adsorption kinetic as a function of the temperature; Figure S2: Superposition between the cyclic voltammogram is of the mixture between 0.015 mM vitamin B12 and an excess of AC in air-saturated 0.10 M potassium phosphate aqueous buffer, pH 7.0. Potential scan rate 20 mV s−1 at t = 0 min (red) with the cyclic voltammogram of vitamine B12 (green) by applying constant potentials between −1.3 and +1 V; Table S1: Parameters obtained from kinetic modelling for different pH.
