# Supplementary material for: Adsorption of Vitamin B12 on Sugarcane-Derived Activated Carbon: Fractal Isotherm and Kinetics Modelling, Electrochemistry and Molecular Modelling Studies
Source: Molecules. 2025 May 8;30(10):2096. doi: 10.3390/molecules30102096 (PMC12113871; doi:10.3390/molecules30102096)
Supplement: Supplementary file 1 [file molecules-30-02096-s001.zip › molecules-3513929-supplementary.pdf]

Supplementary Materials

Table S1. Parameters obtained for kinetic modelling of VB12 adsorption on AC for different initial pH (T= 25°C, mAC= 5 mg, [VB12] = 25mg.L^{-1}).

Model	Parameters	pH=2	pH=4	pH=6	pH=8	pH=10
Pseudo order 1	qe exp	154.2	177.5	184.4	173.8	177.9
	qe the	151.3	170.2	175.8	167.5	169.9
	k1	0,7	0.7	1.18	0.6	0.8
	SSR	1172.0	1172.0	1884.2	1318.2	2367.6
	ARED	5.0	3.8	3.5	5.2	5.0
	AICc	92.2	103.5	115.9	106.6	121.8
	R^2	0.90	0.97	0.90	0.98	0.95
Pseudo order 2	qe exp	154.2	177.5	184.4	173.8	177.9
	qe the	159.8	179.9	184.9	177.4	178.6
	k2	0.006	0,005	0.01	0.005	0.007
	SSR	804298.7	2061667.7	575711.1	1100826.8	1186897.1
	ARED	80.0	121.9	40.1	87.9	57.8
	AICc	273.3	297.8	264.6	281.5	272.7
	R^2	0.99	0.99	0.98	0.99	0.99
Elovich	qe exp	154.2	177.5	184.4	173.8	177.9
	qe the	142.3	179.6	178.4	155.2	165.0
	α	6816.5	589.4	27301.9	6180.3	13141.2
	β	0.06	0.03	0.06	0.06	0.06
	SSR	$4.7.10^{-7}$	5.3	0.006	12266.7	5.10^{-8}
	ARED	13.6	1.6	4.0	9.8	7.9
	AICc	-456.6	-31.5	-209.8	167.2	-514.8
	R^2	0.82	0.97	0.92	0.81	0.87
BS (n,a)	qe exp	154.2	177.5	184.4	173.8	177.9
	qe the	156.3	200.7	184.5	171.4	174.1
	n	1.27	3.3	0.8	1.1	0.8
	τ	1.42	0.8	1.3	1.7	1.6
	α	0.72	1.1	0.4	0.7	0.5
	SSR	0.2	5.3	371843.7	3.41	0.2
	ARED	0,21	1.6	25.5	0.02	0.06
	AICc	-117.9	-31.5	258.7	-42.9	-110.0
	R^2	0.99	0.99	0.99	0.99	0.99

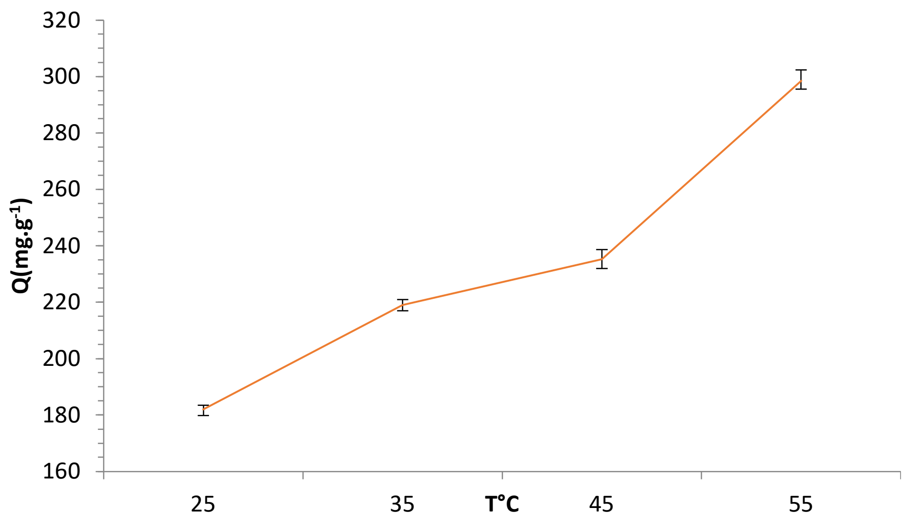

Figure S1. Maximum adsorption capacity obtained for VB12 adsorption kinetic for different temperatures (mAC= 5 mg, Ci= 25 mg.L-1, pH =6, T°C = 25°C, 35°C, 45°C and 55°C.

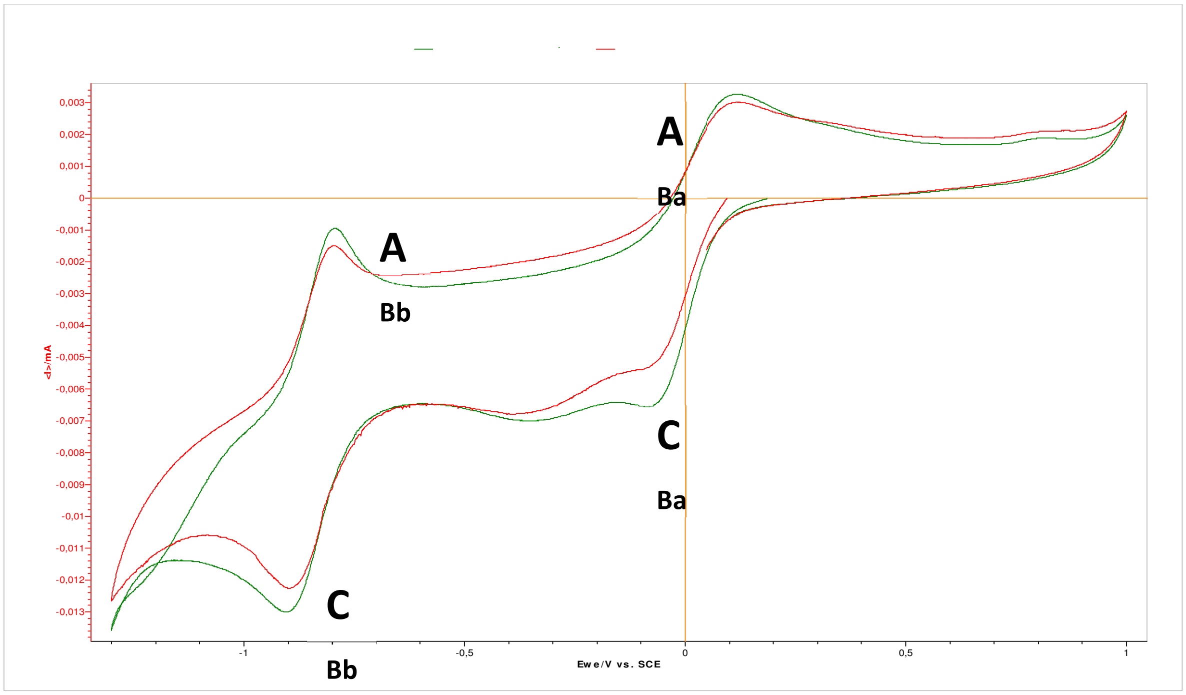

Figure S2. Superposition between the cyclic voltammogram is of the mixture between 0,015 mM vitamin B12 and an excess of AC in air-saturated 0.10 M potassium phosphate aqueous buffer, pH 7.0. Potential scan rate 20 mV s^{-1} at t=0 min (red) with the cyclic voltammogram of vitamine B12 (green) by applying constant potentials between −1.3 and +1 V.

.yaml input file used in GaudiMM docking calculations


```yaml
output:
    path: ./cubeAC1_1_00  # Name of the cuboid and number of replica
    name: cubeAC1_1_00
    verbose: True
    pareto: False
    check_every: 0
ga:
    cx_eta: 5
    cx_pb: 0.5
    generations: 200
    mu: 1
    mut_eta: 5
    mut_indpb: 1.0
    mut_pb: 0.5
    population: 100
similarity:
    module: gaudi.similarity.rmsd
    args: [[Ligand], 0.5]
    kwargs: {}
genes:
    -   name: Ligand
        module: gaudi.genes.molecule
        path: ./mol_files/B12_with_H.pdb   #File of VB12 structure
    -   name: Receptor
        module: gaudi.genes.molecule
        path: ./mol_files/AC1_1.pdb    #File of AC cuboid structure
    -   name: Search
        module: gaudi.genes.search
        radius: 65
        precision: 5
        rotate: True
        target: Ligand
        center: [0, 100, 0]
        interpolation: 0.5
    -   name: Torsion
        module: gaudi.genes.torsion
        target: Ligand
objectives:
    -   name: Clashes
        module: gaudi.objectives.contacts
        which: clashes
        weight: -1.0
        probes: [Ligand]
        radius: 5.0
    -   name: Vina
        module: gaudi.objectives.vina
        weight: -1.0
        receptor: Receptor
        ligand: Ligand
```
